# COVID-19 presenting after Elective Off-pump Coronary Artery Bypass Grafting and Lessons Learned

**DOI:** 10.1186/s43044-022-00286-6

**Published:** 2022-06-04

**Authors:** Sudipto Bhattacharya, Ashok Bandyopadhyay, Satyabrata Pahari, Sankha Das, Asim Kumar Dey

**Affiliations:** 1 Department of Cardiothoracic and Vascular Surgery, Peerless Hospitex Hospital & B K Roy Research Centre, 360, Pancha Sayar Road, Sahid Smrity Colony, Pancha Sayar, Kolkata, West Bengal 700094 India; 2 Department of Anaesthesiology, Peerless Hospitex Hospital & B K Roy Research Centre, 360, Pancha Sayar Road, Sahid Smrity Colony, Pancha Sayar, Kolkata, West Bengal 700094 India

**Keywords:** COVID-19, Coronary artery disease, Coronary artery bypass, Off-pump, Case report

## Abstract

**Background:**

Cases of COVID-19 presenting after elective cardiac surgery are rare. Published literature suggests that such cases have a high morbidity and mortality rate. Here, we report a case of COVID-19 presenting after an elective, isolated off-pump coronary artery bypass (OPCAB).

**Case presentation:**

A 65-year-old obese, hypertensive, hypothyroid lady, with moderate left ventricular dysfunction, presenting with unstable angina, tested negative for COVID-19 at admission, having undergone thrombolysis for a recent inferior wall myocardial infarction, at an outside centre, and coronary angiography revealing left main triple vessel disease, developed signs and symptoms of COVID-19, four days after OPCAB. She was diagnosed with moderate COVID-19 infection. Subsequent contact tracing revealed that her husband was suffering from mild COVID-19 infection and was managed in home isolation. Isolation and early supportive management with moist oxygen, steroids, intravenous antibiotics, zinc and vitamin C helped the patient recover. She was followed up at one month, six months, one year and at eighteen months and has been doing well.

**Conclusions:**

A strong clinical suspicion and repeat testing for COVID-19 is required as the diagnosis may often be missed with COVID-19 mimicking the signs and symptoms of post-cardiotomy syndrome. Preferentially dealing with such cases off-pump, thereby avoiding cardio pulmonary bypass-related complications, may improve outcomes. Isolation and early supportive management help. Adequate follow-up is required in all such cases as cardiovascular complications are common, alongside known long-term sequelae, like anxiety, depression, cardio-respiratory complications, venous thromboembolism and even postural orthostatic tachycardia syndrome.

## Background

The epidemic of coronavirus disease 2019 (COVID-19), caused by the severe acute respiratory syndrome coronavirus 2 or SARS-CoV-2 (2019-nCoV) coronavirus, was first reported on December 12, 2019, in Wuhan, China [1]. COVID-19 causes varying clinical characteristics ranging from cough and fever to pneumonia to acute respiratory distress syndrome (ARDS) and shock [2].

Cases of COVID-19 presenting after elective cardiac surgery are rare. Published literature suggests that such patients have a high morbidity and mortality rate. To the best of our knowledge, there has been no previous case report of such a case of COVID-19, presenting after an elective, isolated off-pump coronary artery bypass grafting (OPCAB), with subsequent follow-up data.

## Case presentation

A 65-year-old obese lady (body mass index, BMI of 30.16) with hypertension, hypothyroidism, recent inferior wall myocardial infarction (thrombolysed at an outside centre), moderate left ventricular dysfunction (left ventricular ejection fraction of 38%) with hypokinetic infero-posterior wall on echocardiography, presented with unstable angina, diagnosed as a case of left main triple vessel disease on coronary angiography at another centre. There was no known COVID-19 infection in the family at that stage, and there was no significant travel history. She tested negative for COVID-19 reverse transcriptase polymerase chain reaction (RT-PCR) at admission for elective, isolated OPCAB. Written, informed consent was obtained from the patient for the procedure and further management.

Isolated OPCAB was done via median sternotomy. The surgery was uneventful, wherein she received four grafts off-pump (skeletonised left internal mammary artery to the left anterior descending artery, reversed saphenous vein graft, RSVG to the ramus intermedius and distal left circumflex artery sequentially and another RSVG was done to the posterior descending artery). She was shifted to the intensive therapy unit (ITU) with stable haemodynamics, in sinus rhythm, under elective mechanical ventilation and minimal inotropic supports. She was extubated after 12 h and had good urine output, her drainage overnight was 240 mL, and her post-operative chest X-ray was acceptable. Her intercostal chest drains were removed on the post-operative day 2 with an overall drainage of 510 mL, and she was off inotropic supports. She was haemodynamically stable, in sinus rhythm, maintaining a room air saturation of 98 and was mobilized from day 3.

**Fig. 1:**
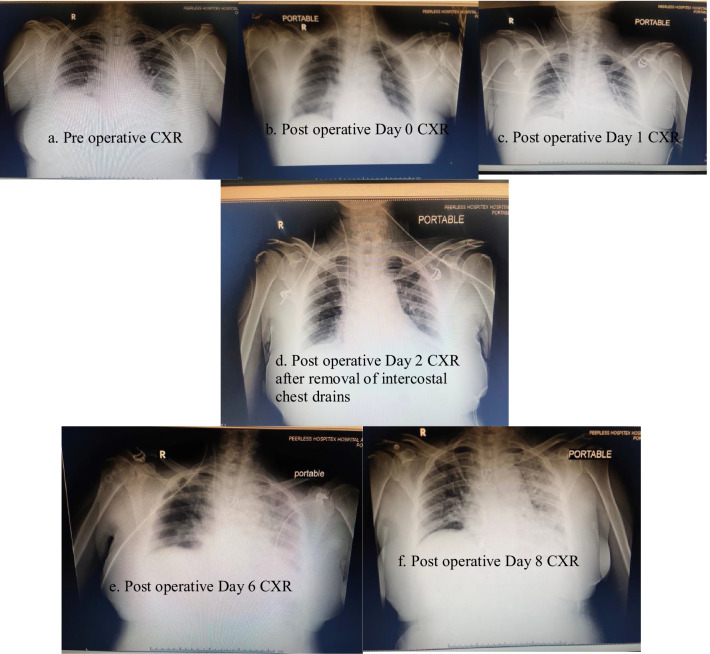
**a**–**f** A collage of chest X-rays (CXR) of the patient during her hospital stay

On day 4, she became febrile and her total leukocyte count (TLC) was raised to 15,100/cmm. Urine was sent for routine examination and it showed numerous pus cells per high power field. Urine culture and blood culture tests were sent. Intravenous antibiotics (Meropenem 1 g thrice daily and Teicoplanin 400 mg once daily) were started and continued for 5 days. She had two episodes of low-grade fever on day 5. Her urine culture revealed no growth after 48 h, and subsequently, her blood culture was also negative. On day 6, she continued having low-grade fever and became dyspneic. Her room air saturation was 93 despite moist oxygen inhalation at 6 L/min. Her chest X-ray showed a hazy left hemithorax. A picture collage depicting her chest X-rays, from the pre-operative stage onwards, is presented in Fig. 1.

A repeat COVID-19 RT-PCR test was positive. The hospital staff underwent the COVID-19 RT-PCR test and came out to be negative. Subsequent contact tracing was done, and the patient’s  husband also tested positive for COVID-19. He was diagnosed as a case of mild disease and managed in home isolation. The institutional management protocol followed, for COVID-19, is shown in Table 1.

**Table 1:**
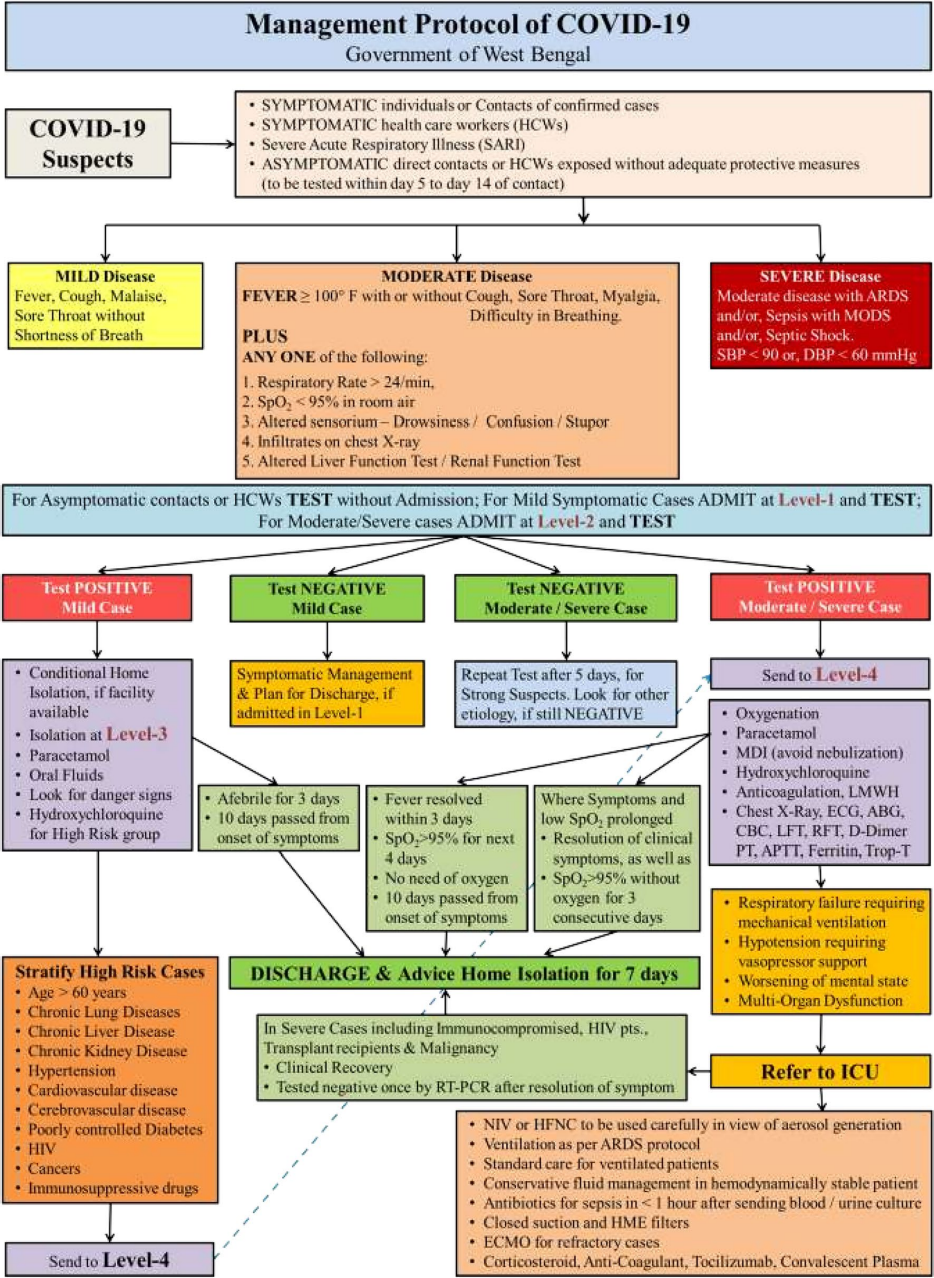

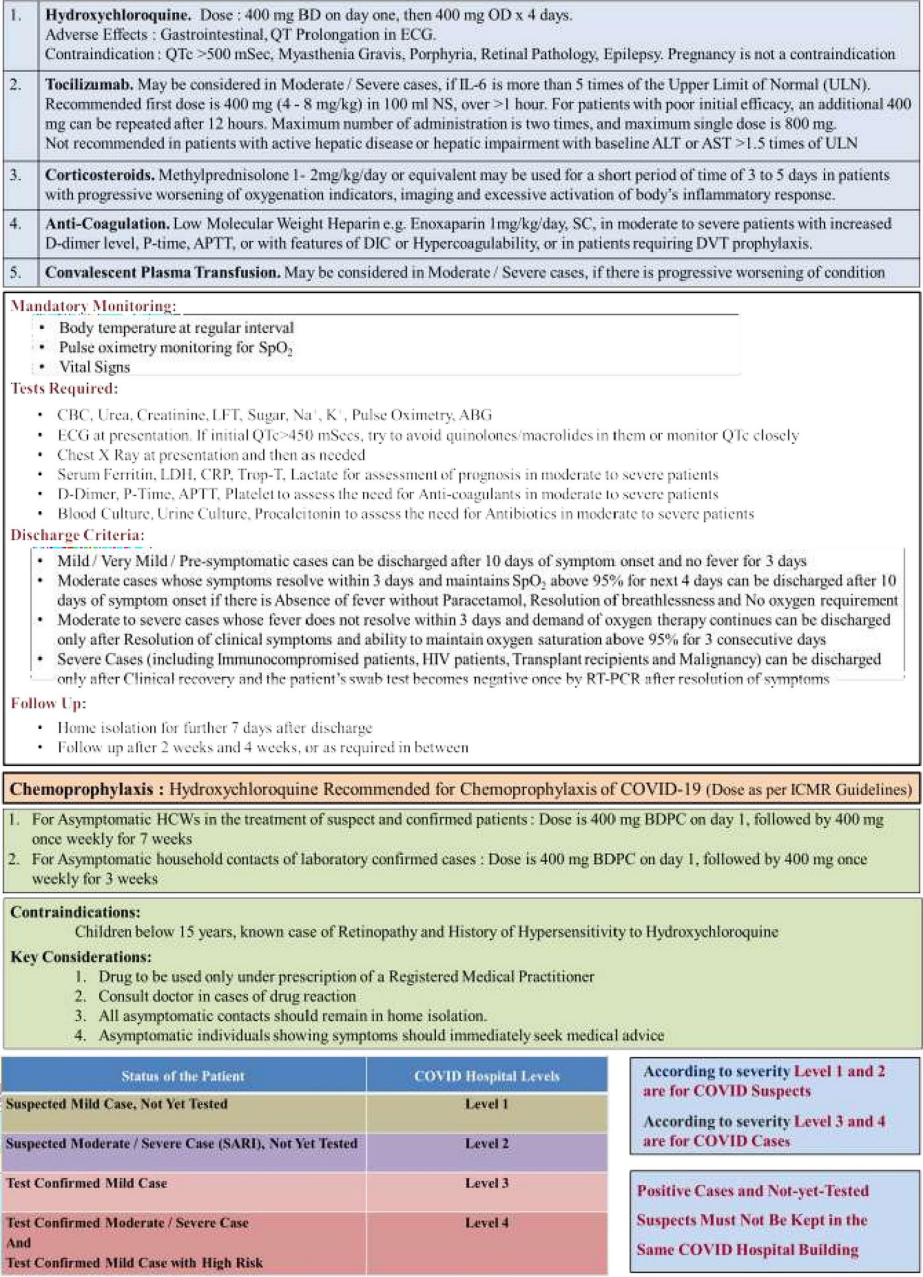
The classification of COVID-19 (mild, moderate and severe disease) and the institutional management protocol, which was followed, both in the case of the patient and her husband, as per the guidelines of the Government of West Bengal, India

Note: Hydroxychloroquine was initially a part of the proposed management protocol in 2020, which was subsequently taken down in 2021. Neither the patient, nor her husband received Hydroxychloroquine as a part of their management in hospital and in home isolation, respectively

**Fig. 2:**
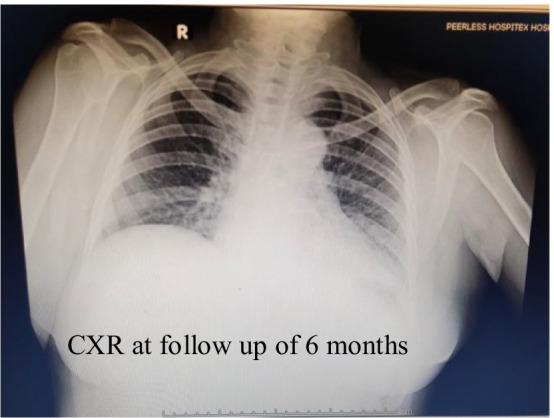
Her chest X-ray (CXR) at a follow-up period of 6 months

The lady was started on oral steroids (Dexamethasone 8 mg daily), zinc (50 mg orally, daily) and vitamin C (orally, 500 mg twice daily after meals) and shifted to the COVID-19 isolation ward under the hospital COVID-19 team of physicians. Her symptoms suggested moderate COVID-19 infection. Her C-reactive protein (CRP) level was 111.9 mg/L, and interleukin-6 (IL-6) level was 78.1 pg/mL. Her computed tomography (CT) chest revealed features suggestive of acute COVID-19 pneumonitis. Medical management continued, and her CRP dropped to 57.9 mg/L, and TLC to 12,200/cmm. Her CRP level dropped further to 18.7 mg/L, and she maintained a room air saturation of 97 at discharge, after two weeks at the hospital. She was subsequently followed up by the cardiac surgical team and the COVID-19 team of physicians at one month, six months, one year, at eighteen months and has been haemodynamically stable, in sinus rhythm, fully ambulant, maintaining a room air saturation of 98. Her follow-up chest X-ray at six months is shown in Fig. 2.

The patient was happy with the way she was managed in hospital and at follow-up and the way her husband was managed during his home isolation period, through Tele-consultation with the hospital COVID-19 team of physicians.

## Discussion

A patient asymptomatic for COVID-19, who tested negative at admission, subsequently turning out to be RT-PCR positive for COVID-19, following surgery, teaches us important lessons about this disease.

Firstly, the incubation period varies. The mean and median incubation period has been reported to be a maximum 8 days and 12 days, respectively [3]. In this case, symptoms suggestive of COVID-19 infection appeared on post-operative days 4 and 5 which led to a repeat RT-PCR test for COVID-19.

Secondly, the RT-PCR test for COVID-19 may not always yield correct results [4]. Even within 4 days of onset of symptoms, some patients may have negative test results.

Again, asymptomatic patients may not always test positive for COVID-19. Also, COVID-19 is notorious for mimicking post-cardiotomy characteristics like fever, dyspnea or a rise in TLC as was evident on post-operative day 4 in the case here. Early diagnosis, isolation and supportive management aided in early recovery.

Any of the above possibilities could hold true for the case in question. She may have been in the incubation period at admission, when she tested negative for COVID-19 on RT-PCR. Or, she was asymptomatic at admission and her initial RT-PCR test yielded an incorrect result. Thus, it was a case of COVID-19, diagnosed late, who underwent OPCAB, was discharged from hospital in two weeks’ time and has been doing well at a follow-up period of eighteen months.

Similar cases of COVID-19 have been reported, who underwent coronary artery bypass grafting, but some perished to ARDS and shock [5], and no follow-up data has been reported for discharged patients [6].

The other reported cases have been done on pump, and cardiopulmonary bypass (CPB) is known to be associated with a systemic inflammatory response, increased red blood cell damage, qualitative and quantitative damage of platelets, raised catecholamine levels, complement activation, protein denaturation, raised extracellular fluid volumes, neurological events like stroke and damaging effects on the heart, kidneys, liver, lungs, etc. Again, non-pulsatile flow, hypothermia, duration of CPB, hypoperfusion and gaseous/particulate microemboli contribute to end organ injury. This case, having been done electively, off-pump, might have helped improve outcome, thereby avoiding known CPB-related complications, which could have been aggravated with COVID-19.

Cardiovascular complications are common post-COVID-19 infection [7]. Patients with acute coronary syndrome, who are infected with COVID-19, often have a poor prognosis. The case in question had recent inferior wall myocardial infarction. Also, COVID-19 infection has known long-term sequelae [8] like fatigue, anxiety, depression, cardio-respiratory complications, venous thromboembolism and even postural orthostatic tachycardia syndrome. Therefore, adequate follow-up is necessary. Rescigno et al. [9], in their case report of a similar patient, suggest concern about adverse outcomes, in patients undergoing cardiac surgery, who might be infected with COVID-19 and underline the importance of additional investigations and experience. Ours has been an attempt in this regard to bridge this gap.

## Conclusions

Cases of COVID-19 presenting after elective cardiac surgery are rare. Most of those reported in the literature have had a high morbidity and mortality rate. Preferentially dealing with such cases off-pump, thereby avoiding CPB related complications, may help improve outcomes. A strong clinical suspicion and repeat testing for COVID-19 is required, as the diagnosis may often be missed with COVID-19 mimicking the signs and symptoms of post-cardiotomy syndrome. Isolation and early supportive management with moist oxygen, steroids, intravenous antibiotics, zinc and vitamin C are beneficial. Here, although we describe this case as COVID-19 presenting post-operatively after an elective OPCAB, a thorough evaluation indicates that the patient was either in the incubation period, when she was tested at admission, or she may have been infected with COVID-19 at admission, but was asymptomatic at that stage, and was false negative on RT-PCR. Thus, she actually may have contracted the virus pre-operatively. She presented late, and with her signs and symptoms mimicking post-cardiotomy syndrome, she was diagnosed only on day 6 following OPCAB, when she underwent a repeat RT-PCR for COVID-19. Subsequent contact tracing showed that her husband was also suffering from mild COVID-19, and he was managed through Tele-consultation. Thus, the case in question was actually a case of COVID-19, who underwent OPCAB. The patient has been followed up, both by the cardiac surgical team and the COVID-19 team of physicians, and has been doing well at a follow-up period of eighteen months. Adequate follow-up is required in all such cases as cardiovascular complications are common, alongside known long-term sequelae, like anxiety, depression, cardio-respiratory complications, venous thromboembolism and even postural orthostatic tachycardia syndrome.

## Data Availability

The datasets used and/or analysed during the current study are available from the corresponding author on reasonable request.

## Abbreviations

ARDS: Acute Respiratory Distress Syndrome
BMI: Body Mass Index
COVID-19: Coronavirus Disease 2019
CPB: Cardio Pulmonary Bypass
CRP: C-Reactive Protein
CT: Computed Tomography
IL-6: Interleukin-6
ITU: Intensive Therapy Unit
OPCAB: Off-Pump Coronary Artery Bypass surgery
RSVG: Reversed Saphenous Vein Graft
RT-PCR: Reverse Transcriptase Polymerase Chain Reaction
SARS-CoV-2: Severe Acute Respiratory Syndrome Coronavirus 2
TLC: Total Leukocyte Count
